# 100 Years of Scientific Evolution of Work and Organizational Psychology: A Bibliometric Network Analysis From 1919 to 2019

**DOI:** 10.3389/fpsyg.2020.598676

**Published:** 2020-12-03

**Authors:** Michele K. Sott, Mariluza S. Bender, Leonardo B. Furstenau, Laura M. Machado, Manuel J. Cobo, Nicola L. Bragazzi

**Affiliations:** ^1^Graduate Program of Industrial Systems and Processes, University of Santa Cruz do Sul, Santa Cruz do Sul, Brazil; ^2^Multiprofessional Residency Program in Urgency and Emergency, Santa Cruz Hospital, Santa Cruz do Sul, Brazil; ^3^Department of Psychology, Lutheran University of Brazil, Cachoeira do Sul, Brazil; ^4^Department of Computer Science and Engineering, University of Cádiz, Cádiz, Spain; ^5^Laboratory for Industrial and Applied Mathematics, Department of Mathematics and Statistics, York University, Toronto, ON, Canada

**Keywords:** industrial and organizational psychology, work psychology, organizational behavior, strategic intelligence, bibliometric, science mapping

## Abstract

In this study, we explore a 100 years of Work and Organizational Psychology (WOP). To do this, we carry out a bibliometric performance and network analysis (BPNA) to understand the evolution structure and the most important themes in the field of study. To perform the BNPA, 8,966 documents published since 1919 were exported from the Web of Science and Scopus databases. The SciMAT software was used to process data and to create the evolution structure, the strategic diagram, and the thematic network structure of the strategic themes of the field of WOP. We identified 29 strategic clusters and discuss the most important themes (motor themes) and their relationship with other clusters. This research presents the complete evolution of the field of study, identifying emerging themes and others with a high degree of development. We hope that this work will support researchers and future research in the field of WOP.

## Introduction

Psychology is, first and foremost, a science that deeply investigates the human mind and behavior through different aspects. The first laboratories that studied human behavior were carried out in 1879 by Wilhelm Wundt (1832–1920). A few years later, Hugo Münsterberg (1863–1913), known as the father of Industrial-Organizational (I-O) Psychology, carried out the first researches focused on human behavior in work environments, focusing on individual differences of workers (Alston and Munsterberg, 1914; Giberson, 2015). Work and Organizational Psychology (WOP) is the facet of psychology that studies in-depth organizational climate and culture as well as work teams and their skills and performance (Giberson, 2015). However, the study of people in the business environment only gained strength after the First World War due to studies related to skills and job performance, morals and motivation, and the selection of soldiers teams (military psychology) (Yerkes, 1918), where psychologists dedicated efforts and aroused the interest of organizations. In this sense, WOP and the study on Organizational Behavior Management (OBM) seek to improve organizational performance through better human resource management, creating a synergy between workers and organizations (Geller, 2002).

The drastic changes in work environments toward technological adoptions and process reengineering transform the definition of work. I-O psychology has an important role in the activity analysis and employee development, showing great potential to generate benefits for companies and professionals (Cascio, 1995), and making it possible to understand people, institutions of society and organizations (Nord, 1980). In this sense, WOP globally supports organizational management and improves relationships between organizations and workers. The knowledge about individual differences and personal satisfaction and the study of the different people’s reality and its relationship with a performance at work are important themes for the WOP research field, and have a direct impact on social development, worker commitment and the relationship between company and employee (Gelfand et al., 2017).

Many researchers dedicate efforts to the field of WOP, and several works have studied the relationships between individuals and organizations. Although many studies have reviewed WOP (Billings and Wroten, 1978; Cascio, 1995; Bond and Smith, 1996; Brutus et al., 2010; Gelfand et al., 2017; Schalken and Rietbergen, 2017; Bal et al., 2019) and discussed several characteristics and their impacts for individuals and organizations, no work presents a complete background of the field of study. This review is the first bibliometric performance and network analysis (BPNA) performed in the field of WOP. To do this, the SciMAT (Science Mapping Analysis Software Tool) developed by Cobo et al. (2012) was used. We present and discuss a 100 years of scientific evolution, the strategic diagram and thematic network structures of the most important themes of WOP.

The paper is organized as follows: Second section presents the materials and methods. In third section, the performance bibliometric analysis is presented. Fourth section contains the strategic diagram and thematic network structure of the most important themes (motor themes). Fifth section presents the scientific evolution of WOP, and sixth section presents the conclusion, limitations, and suggestions for further research.

## Materials and Methods

To carry out this BPNA, we used two scholarly electronic databases, namely Scopus and Web of Science (WoS). The following search string related to the field of WOP was used: (“organizational psychology” OR “industrial psychology” OR “industrial and organizational psychology” OR “work psychology” OR “organizational behavior” OR “vocational psychology” OR “personnel psychology”). Such terms were used in the systematic review of Schalken and Rietbergen (2017). We searched for documents in English, that contain any of the search terms in the title, abstract or keywords, in order not to exclude studies unrelated to the theme. Another criterion used was the choice of document type as published original articles, articles in press and reviews. In this research, we used the SciMAT software developed by Cobo et al. (2012), because it is a free software that supports all stages of scientific mapping (Furstenau et al., 2020a; López-Robles et al., 2020). In this stage, with the support of the SciMAT software, we perform preprocessing, network development and data analysis. For the creation of the scientific evolution and diagrams, we considered the frequency of co-occurrence of keywords. Besides, we use the equivalence index to calculate the bond strength and similarity between clusters, and the simple center algorithm was used to detect and clustering the themes (Furstenau et al., 2020b; Sott et al., 2020).

**FIGURE 1:**
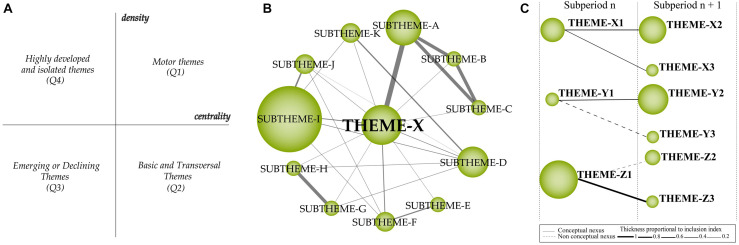
**(A)** Strategic diagram. **(B)** Thematic network structure. **(C)** Thematic evolution structure.

The clusters were plotted in two-dimensional diagrams based on centrality (*x*-axis) and density (*y*-axis) values. The diagram contains four quadrants (Figure 1A), where: Motor themes (Quadrant 1, Q1) are important themes with high centrality and density; Basic and transversal themes (Quadrant 2, Q2) are themes with strong centrality, but low development; Emerging or declining themes (Quadrant 3, Q3) represent themes that need qualitative analysis to understand whether they are emerging or losing relevance; and Highly developed and isolated themes (Quadrant 4, Q4) are clusters with high density and low centrality (Cobo et al., 2012; Furstenau et al., 2020a; López-Robles et al., 2020). Figure 1B shows an example of the thematic network structure performed by SciMAT based on co-occurrence of keywords, where the cluster size represents the number of associated documents and the line thickness represents the strength of the link between the themes (Furstenau et al., 2020b; Sott et al., 2020). Figure 1C provides a good representation of the thematic evolution structure over time (for more information, see Cobo et al., 2012).

We exported 12,015 related documents from Scopus (7,533) and WoS (4,482) databases, which contains a total of 21,298 keywords. In preprocessing, 3,049 duplicate documents were excluded. After, 706 words with the same meaning were grouped, such as “industrial and organizational psychology” and “I/O psychology” among others. Moreover, misspelled keywords have been corrected and irrelevant terms like “article” and “review” have been removed. A total of 8,966 documents and 20,592 words were included for analysis. The period covered in this research was from 1919 until October 11, 2019, because the concept of vocational psychology was first discussed in 1919 (Mitchell, 1919). To explore the scientific evolution of the field of study, we divided the period into four subperiods (1919–1943; 1944–1968; 1969–1993; 1994–2019).

### Performance Bibliometric Analysis of WOP

**FIGURE 2:**
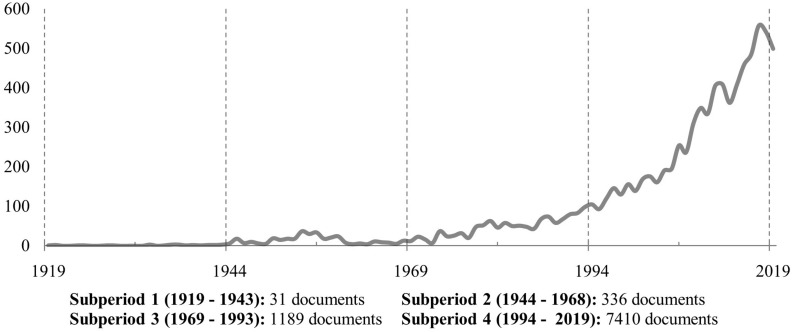
Number of publications over time (1919–October 2019).

One hundred years of scientific evolution of WOP are covered in this article, from the first discussion on vocational psychology by Mitchell (1919). Figure 2 presents the evolution of publications over time. It is possible to observe that in the first decades the theme was little discussed, but the exponential growth shows the valorization of the field of WOP and the efforts dedicated to research related to human behavior in organizational environments. Despite the slow start, the field of study shows its importance reaching hundreds of publications in recent years. The decline in the number of publications in 2019 is due to the date of data collection (October 11, 2019).

Table 1 shows the journals and universities that most published studies related to WOP in the study period. It is possible to see that the Journal of Applied Psychology has the largest number of publications, followed by the Journal of Management Education and the Industrial and Organizational Psychology Journal. It is interesting to note that the universities that most publish researches related to WOP are from the United States, highlighting the importance of other countries and universities to dedicate greater efforts to the field of WOP.

**TABLE 1:** Journals and universities that most published studies related to WOP.

Journals	Doc.	Organizations and universities	Doc.
Journal of Applied Psychology	242	State University System of Florida (United States)	174
Journal of Management Education	236	University System of Georgia (United States)	95
Industrial and Organizational Psychology	211	Pennsylvania Commonwealth System of Higher Education (PCSHE) (United States)	92
Journal of Organizational Behavior	191	University of North Carolina (United States)	89
Journal of Organizational Behavior Management	187	University of California System (United States)	87
Personnel Psychology	125	University System of Maryland (United States)	86
Journal of Vocational Behavior	113	Michigan State University (United States)	77
Journal of Management	109	State University of New York (SUNY) System (United States)	71
Journal of Managerial Psychology	94	Arizona State University (United States)	70
Journal of Occupational and Organizational Psychology	78	University of Nebraska System (United States)	69

Table 2 presents the most cited and productive authors in the period. The authors with the highest number of citations are Schaufeli, Maslach, C. and W.B., Luthans, F. On the other hand, the most productive authors are Luthans, F. with 55 documents, Aguinis, H. (34) and Bakker, A.B. with a total of 25 documents. This analysis allows us to identify the performance of the most important authors in the field of study over time. It is important to note the fact that the 10 most productive authors are male, which may be related to the patriarchal education that for a long time prevented women from studying. However, currently, psychological science is composed of a large number of women, which explains the three female names that appear among the ten most cited authors.

**TABLE 2:** Most cited and productive authors.

Most cited authors	Cit.	Most productive authors	Doc.
Schaufeli, W.B.	8477	Luthans, F.	55
Maslach, C.	5863	Aguinis, H.	34
Luthans, F.	5589	Bakker, A.B.	25
Schneider, B.	4688	Austin, J.	22
Meyer, J.P.	4207	Avey, J.B.	21
Bakker, A.B.	4199	Ashkanasy, N.M.	18
Venkatesh, V.	3367	Schaufeli, W.B.	16
Mitchell, M.S.	3326	Cooper, C.L.	12
Cropanzano, R.	2977	Stagner, R.	10
Gagné, M.	2838	Meltzer, H.	10

### Science Mapping Analysis

In this section, the science mapping analysis of WOP is presented. The strategic diagram shows the most important themes according to their centrality and density. Besides, the themes were measured using bibliometric indicators such as core documents, h-index and citations (see Table 3). The thematic network structure provides a good depiction of the co-occurrence of themes. The thematic evolution structure undercovers the most significant themes and reveals how the field of study is advancing overtime.

**TABLE 3:** Bibliometric indicators used in this research.

Bibliometric indicators	Description
Centrality (C.)	Centrality (C.) represents the amount of co-occurrence (links) of a cluster with other clusters surrounded. The C. provides a good representation of the importance of a theme to the entire field of research. In other words, the theme is vital for anyone interested in the area.
Density (D.)	Density describes the development of a cluster in the field of research. The more the clusters appear together in documents stronger will be the thickness of connections. The D. describes the capacity of themes to endure over time in the field of research.
Core documents	Core documents is a mapper parameter from SciMAT that returns documents present in which at least two nodes.
h-index	In this case, the h-index measures the maximum value of h that the given theme has been cited at least h times.
Citations	The citation indicator indicates the number of times that a particular theme has received. This indicator is a reasonable way to identify of the overall quality of themes.

### Strategic Diagram Analysis

**FIGURE 3:**
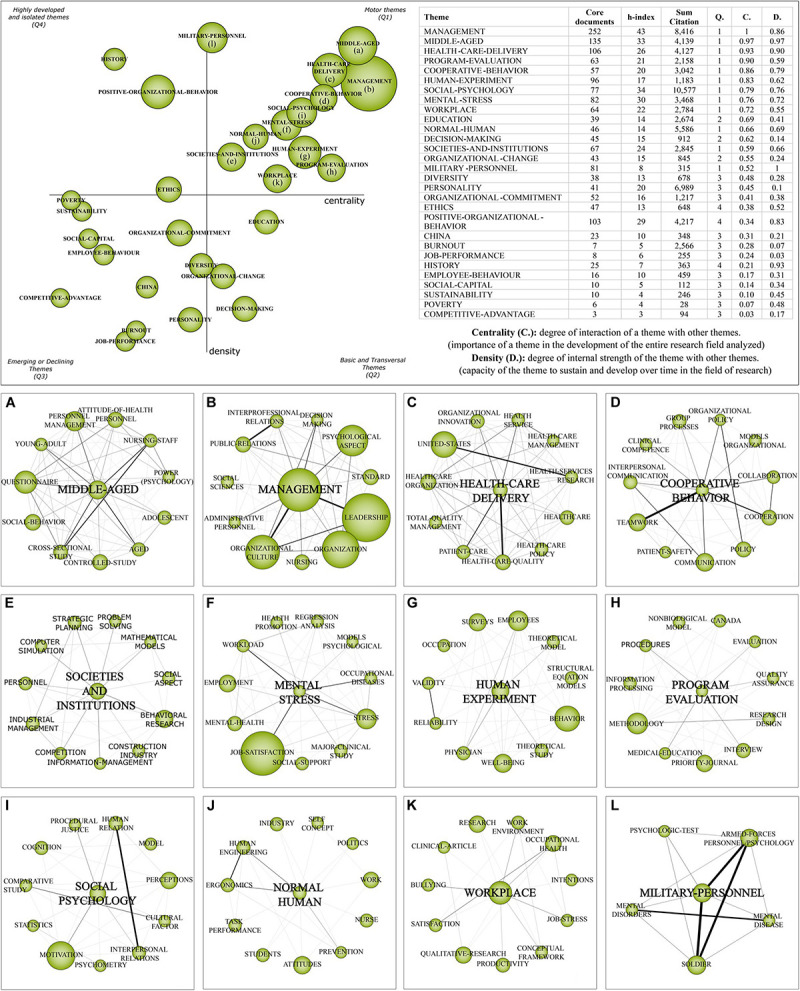
Strategic diagram and thematic network structure of the motor themes (1919-2019). **(A)** middle aged, **(B)** management, **(C)** healthcare delivery, **(D)** cooperative behavior, **(E)** societies and institutions, **(F)** mental stress, **(G)** human experiment, **(H)** program evaluation, **(I)** social psychology, **(J)** normal human, **(K)** workplace, and **(L)** military personnel.

With the support of the SciMAT software, we create a strategic diagram of the field of study. Figure 3 presents 29 strategic clusters related to WOP and the level of development of each cluster through the core documents, h-index, sum citation, quadrant (Q), centrality (C), and density (D). It is possible to note that twelve (12) clusters are motor themes with high development, three (3) clusters are basic and transversal themes, eleven (11) are emerging or declining themes and three (3) are highly developed and isolated themes. Also, Figure 3 shows the thematic network structure of the motor themes to discuss the relationships of the most important clusters with other themes.

Although the focus of this paper is to discuss the motor themes (Q1), we can observe the development of other strategic themes in the field of WOP (Figure 3). Basic and transversal themes (Q2) are related to education, organizational change and decision-making. Positive organizational behavior, history and ethics are highly developed and isolated themes (Q4), these efforts represent the use of history and cultural knowledge to promote actions and organizational changes. Q3 consists of eleven emerging or declining themes and presents clusters related to different WOP approaches. On the one hand, this quadrant shows concerns with workers, such as Burnout syndrome, job performance and personality clusters. On the other hand, it presents research related to organizational development, such as competitive advantage and organizational commitment.

### Thematic Network Structure Analysis

In this subsection, we present the thematic network structure of the motor themes of the strategic diagram (Figure 3). The ‘MIDDLE-AGED’ cluster (Figure 3A) is highly developed and dense and has 135 core documents. The relationship of this cluster with the sub-themes elucidated the challenges that groups of workers of different ages face in the labor market, the different attitudes, needs and skills of these professionals to carry out similar or different activities. Discussion related to middle-aged workers addresses working conditions, employee perspectives and the relationship of workers with organizational processes. Work in stressful environments directly affects the physical and mental health of workers, and WOP seeks to understand the variables that affect workers psychologically. Research related to middle-aged and older workers also seeks to prove the effectiveness and stability of these professionals (Meier and Kerr, 1976). This cluster is related to the subthemes ‘ADOLESCENT’ and ‘YOUNG-ADULT’ and highlights research related to workers of different ages. This topic is discussed in different scenarios, from times when older people were excluded from the labor market to the acceptance and appreciation of these people due to their professional experience, and the difficulty of certain groups in maintaining stability at work. In addition, the constant changes in the world of work and the recent digital transformation lead to new challenges related to inclusion, training, and skills. The sub-themes ‘QUESTIONNAIRE’ and ‘CONTROLLED-STUDY’ represent the main research instruments and research types related to this theme. Generally speaking, this cluster represents the concern with the inclusion of professionals of different ages in organizations, and related research discusses team management, individual attitudes and social behavior.

The ‘MANAGEMENT’ cluster (Figure 3B) has the largest number of core documents (252), and the most important subthemes related to management are ‘ORGANIZATIONAL-CULTURE,’ ‘LEADERSHIP,’ and ‘PSYCHOLOGICAL-ASPECT.’ This cluster addresses the relationship between psychology and management over a 100 years of WOP. While the themes of organizational culture and leadership are related to organizational issues and the influence of organizational on individuals, the ‘psychological-aspect’ cluster takes the opposite path by studying how individual characteristics affect the structure, values and culture of organizations, in order to understand how the psychological dimensions of individuals and their subjectivities interfere and influence organizational processes and management. Due to problems related to human resources management, companies started to recognize variables related to human characteristics and started looking for other ways to manage the company and consider the knowledge and skills of individuals as a competitive advantage (Anderson et al., 1994). In this sense, the organizational culture is a set of values that guides the company (Schneider et al., 2013) and is directly related to management, behavior and organizational performance (Goldman et al., 2006). Likewise, the organizational climate is an important factor that transforms workers’ experiences in the work environment (Schneider et al., 2013). Management and traditional leaderships are changing due to the constant changes in the world of work and the demands of organizations and social development. This transition creates new challenges for workers, who need to adapt to complex organizational demands (Lichtenstein et al., 2006), and for leaders who need to constantly change to deal with the dynamism of organizations and individuals (Dorfman et al., 1999). The relationship between management and WOP shows the need to consider workers as biopsychosocial individuals, with biological predispositions, and subjects of a unique constitution derived from their social history, culture and environment.

‘HEALTHCARE-DELIVERY’ (Figure 3C) is a motor theme with high density and centrality. The subtheme with the largest number of associated documents is ‘UNITED-STATES’ because the country is the largest producer of research in the field of study and has dedicated research efforts related to healthcare delivery. The provision of health services is a challenge because it requires knowledge about the individual needs of patients that go far beyond the technique. The main discussions in this cluster are related to quality improvement, mainly to enhance processes and quality of care, and to transform the organizational behavior of health service companies (Jessee, 1981). Historically, health service delivery teams were unidisciplinary and with limited knowledge (West and Lyubovnikova, 2013), however the transformation of the world of work requires multidisciplinary teams to provide adequate care to patients, and to cope with challenges related to the modernization of work.

The ‘COOPERATIVE-BEHAVIOR’ cluster (Figure 3D) has 57 core documents and the main associated subthemes are ‘COMMUNICATION,’ ‘TEAMWORK,’ and ‘COOPERATION.’ This theme highlights the development of researches to investigate organizational behavior to achieve organizational results. In this sense, organizations seek to understand human relationships and the subjectivity of individuals to achieve collaboration toward personal and organizational goals (Håkonsson et al., 2016). Discussions about cooperative behavior focus to understand the importance of teamwork, cooperation and organizational communication because perfect integration between team members provides many individual and organizational advantages. For this, communication allows managing the team’s knowledge, experiences and motivation in the same direction as the organizational objectives, and WOP seeks to understand and enhance individual skills for employee satisfaction and organizational productivity.

The ‘SOCIETIES-AND-INSTITUTIONS’ cluster (Figure 3E) has 67 core documents and the related works address different characteristics of societies and institutions and how they affect and are affected by individuals. Societies are networks composed of people and institutions are integrated by processes and individuals. The main related subthemes are ‘BEHAVIORAL-RESEARCH’ and ‘STRATEGIC-PLANNING,” these concepts show the need to consider the characteristics of workers in the organization’s strategic planning, as well as considering individual knowledge as strategic for solving problems and competitiveness. The theme ‘behavioral research’ is associated with in-depth studies on human behavior in organizations. In addition, this cluster appears related to the theme ‘societies-and-institutions’ because it studies how the behavior of individuals affects, transforms, influences, and is influenced by society and organizations over time. Research related to organizational behavior has helped organizations to understand the cognitive capacity, personality, attitudes and leadership (Ilies et al., 2006).

‘MENTAL-STRESS’ (Figure 3F) has strong density and centrality and 82 core documents. This theme has a strong relationship with the subthemes ‘JOB-SATISFACTION,’ ‘STRESS,’ ‘EMPLOYMENT,’ and ‘OCCUPATIONAL-DISEASES’ among others. It is possible to note that this cluster is focused on the worker and on factors that cause stress and worker satisfaction because job satisfaction is also associated with better mental health conditions. In this sense, studies seek to understand the physical, psychological and behavioral responses of employees in stressful work environments, which can trigger mental exhaustion or Burnout syndrome that is characterized by emotional exhaustion, depersonalization and low personal fulfillment (Crawford, 1993). Burnout is emotional exhaustion due to work, which arises as a response to chronic stress and interpersonal stressors resulting from the work environment. Burnout can be defined by three dimensions: exhaustion, cynicism, and a sense of ineffectiveness. The tension derived from stress causes divergences between the worker and work (Maslach, 2003). The last 25 years of research have contributed significantly to a better understanding of the complexity of Burnout, the studies have expanded internationally, which has resulted in new conceptual models. In this way, new interventions are proposed to provide relief from exhaustion in the world of work, which contributes to people’s health and well-being (Maslach et al., 2001). Workers’ health and workplace stress are being extensively studied by researchers over time (Szilagyi and Holland, 1980; Cooper et al., 1989; Ilgen, 1990; Schaufeli and Peeters, 2000; Luthans, 2002; Bakker et al., 2007; Gagné and Bhave, 2011; Mitchell et al., 2019), showing the importance that organizations are giving to the theme. In this context, the organizational psychologist can assist in the promotion and prevention of workers’ mental health.

The ‘HUMAN-EXPERIMENT’ cluster (Figure 3G) has a strong relationship with ‘BEHAVIOR,’ ‘EMPLOYEES,’ ‘WELL-BEING,’ and ‘OCCUPATION’ subthemes, among others. The ‘human experiment’ term derives from Taylor’s scientific management worker selection models. In this phase, laboratories were built to select and train workers. Currently, this term is used in Psychology in a more restricted way. Works related to this cluster investigated issues related to personality (Belwalkar and Tobacyk, 2018; Gøtzsche-Astrup, 2018), leadership (Rast et al., 2016; Scott and Pringle, 2018; Madrid et al., 2019), social assistance (Engstrom, 2019) and organizational justice (Lyu, 2016) among others. The variety of research related to this topic highlights the efforts of researchers to understand human behavior in work environments.

The theme “PROGRAM-EVALUATION” (Figure 3H) has 63 core documents and is related to the subthemes ‘METHODOLOGY,’ ‘EVALUATION,’ and ‘INTERVIEW’ among others. This cluster discusses data collection and analysis for the assessment of organizational scenarios, in order to understand the efficiency and effectiveness of its sectors and to find ways to improve management and work teams. This cluster presents research related to interventions in the workplace to improve social capital (Meng et al., 2019), delivery care assessment (Dainty et al., 2018), emotional intelligence of managers (García et al., 2017), motivation and performance of health volunteers (Li et al., 2014), and teamwork improvement intervention (Clay-Williams and Braithwaite, 2015). In this sense, this cluster contains research that evaluates and processes information about procedures, quality and performance in the workplace.

The ‘SOCIAL-PSYCHOLOGY’ cluster (Figure 3I) expresses relationships with the ‘MOTIVATION,’ ‘INTERPERSONAL-RELATIONS,’ and ‘HUMAN-RELATIONS’ subthemes, among others. These subthemes, have great importance in the organizational context, have social determinants and are objects of study of Social Psychology. Fiedler (1954) reported different studies on interpersonal relationships and teamwork. Likewise, Lichtman and Hunt (1971) presented several theories that discuss organizational behavior and concluded that unilateral normative approaches are more restrictive and less useful for the broad understanding of the individual and behaviors in the workplace. Social Psychology studies the individual and relations with the environment, enabling the understanding of organizational behaviors and attitudes that are based on the social environment. The insertion of the psychologist in different contexts allows the construction of interdisciplinary studies (Guastello, 2011) and covers human diversity and multiple factors relevant to the construction of the individual’s personal, social and professional identity. The expansion of Organizational Psychology led to the migration of several professionals from Social Psychology to the business area. In United States, the faculty of business universities has been integrated by many social and organizational psychologists (Staw, 2016). In addition, there are relevant differences in research in Social Psychology and Organizational Psychology that need to be considered when thinking about the training of qualified professionals to work in the organizational context (Stoker, 2012). Thus, perception and cognition are internal factors that directly affect the motivation of the worker; while cultural factors and human/interpersonal relationships are external factors that the subject appropriates to symbolize and create a repertoire of values. This explains the ambiguity of perceptions that workers can present about the same stimulus, because the individual and intelligence, as stated by Guastello (2011), are composed of three components: convergent thinking (logical, rational), divergent thinking (creativity) and the ability to learn from an individual or other people’s experience.

The ‘NORMAL-HUMAN’ (Figure 3J) cluster has 46 core documents and is related to the ‘ATTITUDES,’ ‘HUMAN-ENGINEERING,’ and ‘ERGONOMICS’ sub-themes, among others. This cluster discusses issues such as security effectiveness (Rosenbloom et al., 2009), organizational justice (Jordan and Turner, 2008), team’s perception of leadership decisions (Morris et al., 2000) and organizational behavior management (Milne and Kennedy, 1993), among others. Through this cluster, it is possible to observe efforts in research on actions and perceptions of individuals in organizational environments, these studies allow to understand the attitudes of individuals in an attempt to try to predict behaviors and integrate people in the best possible way. The term ‘NORMAL-HUMAN’ has been used for a long time to separate individuals who showed physical or mental differences from those considered ‘normal.’ There was a paradigm of normal thinking and human behavior, which was accepted in social and organizational contexts. Thus, many studies still use this term, which is however not considered adequate to refer to individual differences.

The ‘WORKPLACE’ theme (Figure 3K) has 64 core documents, mainly related to the ‘WORK-ENVIRONMENT,’ ‘OCCUPATIONAL-HEALTH,’ ‘JOB-STRESS,’ and other subthemes. The focus of workplace-related research is to understand the impact of the work environment on workers’ health, satisfaction and engagement. Authors addressed stress management strategies (Joseph et al., 2019), job satisfaction and performance (Pang and Ruch, 2019), work motivation and quality of work-life (Kocman and Weber, 2018), leadership team behaviors (Yanovsky et al., 2014) and organizational socialization (Cooper-Thomas et al., 2011) among others. Research related to the workplace continues to increase due to organizational changes, such as industrial revolutions that alter the organizational processes and skills required of workers.

The ‘MILITARY-PERSONAL’ (Figure 3L) theme was widely discussed in times of war and post-war, to understand the skills needed to form a good team of soldiers. This cluster has a strong relationship with the subthemes ‘PSYCHOLOGIC-TEST’ and ‘MENTAL-DISORDERS,’ it is also related to the ‘SOLDIERS’ and ‘ARMED-FORCES-PERSONNEL’ subthemes that represent the research focus of this cluster. Dated mainly from the war and post-war periods, the related studies addressed variations in the personality of soldiers (Nyman, 1956), emotional status (Robbins, 1956), suitability for military service (Brodman et al., 1954) and psychiatric aspects (Ostwald and Reid, 1954; Zuring, 1954; Smith, 1955). It is possible to perceive that emergency scenarios of wars were factors that stimulated research in the psychological area, mainly related to the understanding of individuals in extreme environments.

### Scientific Evolution Structure Analysis

**FIGURE 4:**
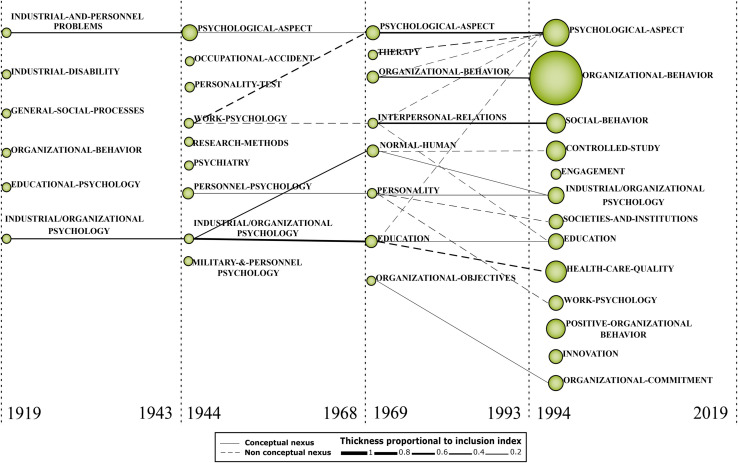
Evolution of thematic areas.

With the support of the SciMAT software, we created an evolutionary map of 100 years of WOP (Figure 4). The map allows us to analyze the most important clusters throughout the subperiods and the relationships with other themes. The cluster size is proportional to the number of documents related to each theme, and the lines represent the connection between the themes, where thick continuous lines represent strong connections between themes from different subperiods (Cobo et al., 2012). The first subperiod (1919–1943) has a few clusters due to the number of documents found. In this subperiod, it is already possible to observe the importance of themes such as organizational behavior and industrial and personnel problems. However, in this period the studies were still isolated and with little representativeness and have no connection with the next subperiod. In the second subperiod (1944–1968) it is possible to see the expansion of the field of study and the spread of WOP in different contexts, such as military, psychiatry, personality tests and accidents at work. In this subperiod, the theme ‘PSYCHOLOGICAL-ASPECT,’ understood as psychological factors that influence behavior, solidified in the field of study and continued to be discussed in the next subperiods, also, it started to have a strong connection with organizational objectives.

In the third subperiod (1969–1993), other themes are discussed, such as therapy, education and interpersonal relations, showing the expansion of the field of WOP and the transformations in the world of work. In this subperiod, all themes are related to the next subperiod. The organizational objectives theme represents organizational goals in all aspects, from the expected profit, to social relations and organizational culture. This theme is strongly linked to organizational behavior in the last subperiod, while interpersonal relations have a very strong relationship with social behavior. Finally, the fourth subperiod (1994–2019) is composed of themes from previous periods that remained strong and with great relevance in the field of WOP, and themes that were built from themes from previous periods such as societies and institutions, healthcare quality and engagement, among others. In addition, new themes unrelated to previous periods have emerged, such as positive organizational behavior, innovation and organizational commitment. The transformation of themes and the emergence of new clusters in recent years show the expansion of the field of study and the transformation of the world of work overtime.

### Analysis and Discussions of the First Subperiod (1919–1943)

Psychology, which for many years was linked to philosophical thinking, was considered a scientific discipline only since 1879. At the Sorbonne Institute of Psychology, considered the birthplace of specialists in industrial psychology, the teaching of applied psychology was included only in 1920 (Bonnardel, 1949). Initially anchored on a strong experimental basis, industrial psychology was the basis for formulating several approaches that sought to explain human behavior, such as Structuralism, Behaviorism, Psychoanalysis, Gestalt, Humanist Schools, Social Psychology, among others. In this subperiod, the world still felt the consequences of the First World War (1914–1918), and the world industry was looking for ways of growth and recovery. In 1919, Mitchell held the first discussion on the application of psychology in different contexts. Mitchell (1919) argues that educational, vocational or industrial issues were experienced differently by each individual and in these contexts the focus of psychology was problematic issues or the expected result and not the individual, and in this sense, Mitchell defended the greater relevance of clinical psychology.

From 1920 onward, studies related to the contributions of Industrial Psychology gained visibility. While authors like Link (1920) defended the use of psychology in the industrial context as a way of solving problems, Brierley (1920) discussed the difficulty in understanding the ways of thinking and the motivations of workers, as well as the influence of the existing social conflict. As shown in Figure 4, the main issues raised by studies from this period referred to the social processes that impacted the lives of workers and influenced the effectiveness of work, and industrial and personnel problems. At this time, strongly marked by Behaviorism, the performance of industrial psychology was focused on professional issues, such as the application of tests for personnel selection and verification of skills and studies related to productivity. In this sense, in 1927 the first work appears that shows concern with the mental health of the workers and with ways to prevent occupational injuries (Knight, 1927) denoting the beginning of research on more subjective issues such as communication, motivation and human behavior.

### Analysis and Discussions of the Second Subperiod (1944–1968)

In this subperiod, several studies were carried out related to WOP. This is because the Second World War (1939–1945) spurred several changes in society and favored changes in labor processes and relations (Hilgard, 1946). In this subperiod, the terms industrial psychology, work psychology and organizational psychology appear, initially without a clear distinction between the concepts, which ended up being used interchangeably. Although there is no consensus on these terminologies, it is worth presenting a conceptual definition of these psychological aspects:

• Industrial psychology refers to the “study of human behavior in aspects of life related to the production, distribution and use of goods and services of our civilization” (Tiffin and Mccormick, 1975).
• Organizational psychology is defined as “applying knowledge from psychological science to issues related to human work, to promote workers’ health and job satisfaction” (Goulart, 1998).
• Work psychology uses concepts, models and methods from psychology to describe and understand the behavior of individuals in the organization, seeking to “meet the needs of workers, without ever forgetting to increase the company’s benefits and income” (García et al., 2003).

In the early years of this subperiod, Bennett (1948) pointed to the large number of psychologists working in the industrial context and the increase in the prestige of the profession. The author explained this change due to the increased concern of industrial managers with human elements, the failure of businesses that used arbitrary and paternalistic methods to maintain worker efficiency, and the ease shown by psychologists to solve problems, especially during the Second World War. Although some managers were skeptical about the value of the psychologist’s insertion in the industry (Colmen, 1954), and others showed the fear that workers would understand this inclusion as something negative and invasive, Bonnardel (1949) pointed out that:

in many cases these services have been supported by delegations of workers who have understood the important role which the psychologist can play in bettering the conditions of workers, in achieving a more equitable distribution of jobs, and in giving to each every chance for advancement which his basic qualities make him deserve (Bonnardel, 1949, pp. 51–52).

With the increase of psychologists in the industry, new questions were raised, such as psychological aspects (Bennett, 1948) and other themes represented in Figure 4. Psychological aspects (which range from psychic processes of thought, perception, memory, emotion, and mood states), along with physical, social and organizational aspects, are considered the resources of work, and contribute to the understanding of how work impacts the well-being and performance of workers (Van Veldhoven et al., 2020). Shartle (1950) pointed to the growing number of didactic books on industrial and personal psychology, human relations, social issues and psychological aspects of labor relations. Psychological aspects were also important during the application of personality tests (Rodger, 1947; Giese, 1948; John, 1948; Clark, 1949; Kates, 1950), which gained visibility after the world’s knowledge about the massive use of American army during World War II (Bonnardel, 1949). At the industrial level, personality tests were used to promote a more efficient staff selection, to reduce factory costs (Giese, 1948). Several studies on the effectiveness, applicability and reliability of these tests were also carried out in this subperiod because although the application has gained strength, the authors considered it relevant to carry out experimental studies that proved the efficiency, as well as to evaluate the psychological factors that led to certain behaviors or responses (Newman, 1954; High et al., 1955; Astin, 1958).

### Analysis and Discussions of the Third Subperiod (1969–1993)

In 1969, Campbell said that “the day of the industrial psychologist has arrived,” reporting that human issues have assumed great relevance in large organizations and in the thinking of many organizational managers, making these spaces conducive to the psychologist’s work. However, the author questions the training of these professionals, who leave the academy with theoretical knowledge, and often, for not being able to put the knowledge into practice, they end up giving way to professionals from other areas. Campbell reiterated the need for professional qualification and practical knowledge for a more emphatic role of the psychologist in organizations (Campbell, 1970). In addition, a variety of possible actions emerged for the psychologist, relating to teaching in fields such as management and administration (Perloff, 1992; Schippmann et al., 1992) and linked to gender discussions (Kennedy, 1991; Studd and Gattiker, 1991; Tenopyr, 1992) because care professions, such as Psychology, are historically attributed to women.

In this subperiod, discussions on psychological aspects remain relevant, gaining greater depth and enabling an expanded view of the individual. Hofkosh (1970) mentioned that organizations improve and combine the efforts of workers, directing them to a specific purpose. The author argued that conflicts between personal and organizational needs can occur, because each individual has their demands and needs. However, when composing a workgroup, each individual suffers a certain level of depersonalization and deindividualization, which is necessary for group fusion and interpersonal relationships. In this way, human relations, already mentioned in some works in the previous period (Shartle, 1950; Sartain, 1951) gain greater prominence with discussions regarding interpersonal relationships and are more satisfactory in small companies, where workers can move around by the groups. Shartle (1950) considers that human relations are more satisfactory in small companies, where workers can move around by the groups, because in larger organizations, there is a tendency for rigidity in work processes and greater complexity in communication, requiring a higher level of de-individualization for compliance with organizational objectives (Hofkosh, 1970). In this sense, Farris et al. (1973) mentioned the importance of individuals’ interpersonal trust and discussed the socializing effects of organizational experiences and the different behaviors assumed in different organizational climates. For the authors, these factors influence the level of confidence, which influences and is influenced by the current cultural norms. Dailey (1980) argues that behavioral phenomena are influenced by the team’s cohesion and size, and mainly, by the way leaders act and delegate tasks.

In this subperiod, many discussions about non-industrial organizational contexts started, as related to the education and administration of educational organizations (Bogue, 1969; Inbar, 1980), continuing education, teaching methods and educational programs for staff development (Hofkosh, 1970; Gallos, 1993; Golden-Biddle, 1993; Thompson, 1993), behavior management in educational organizations (Maher, 1981, 1985), the possibility of conducting group therapy in schools (Litvak, 1991), and the inclusion of minority groups in the classroom (Shallenberger, 1991) or in specific schools (Selinske et al., 1991). Personality studies were also carried out (Barrick and Mount, 1991; Stumpf and Dunbar, 1991), organizations related to health care, with discussions on stressful sources (Cooper et al., 1989; Crawford, 1993), improvement of work performance (Makin and Hoyle, 1993), disease prevention (DeVries et al., 1991), ethics (Goffee, 1993; Goss, 1993) and mental health (Wright et al., 1993).

### Analysis and Discussions of the Fourth Subperiod (1994–2019)

Organizational behavior, which was an emerging theme in the third subperiod, appears as a motor theme in the fourth subperiod, with a significant number of studies carried out. Rousseau (1997) defended a new organizational era, with more competitive organizations and expansion of traditional concepts. In this context, organizational behaviors also change and adapt, making research on organizational behavior assume an increasingly important role, especially about the health and well-being of workers (Wright and Cropanzano, 2000). Wilpert (1995) analyzed several publications that argued that organizational phenomena are socially constructed through interaction between actors. These social constructions are the rituals, stories, myths, structures or objectives that form the organizational basis and the set of shared values. In this sense, organizations are formed by a network of meanings, which are shared in an intersubjective way and are maintained by communication processes and daily social interaction. In this sense, Iuscu et al. (2012) consider it important to understand organizations as rational entities, as open systems formed by coalitions of power groups, in which the processes are not watertight. Thus, factors such as the motivation of the groups and the feeling of belonging to the organization are directly impacted by the types of organizational leadership. In addition, Eldridge and Nisar (1994) argued that culture influences behavior and management practices. Larson and Benson (1994) mentioned that “cultural issues that have an impact on the social and business components of organizational life,” while Carlisle and Manning (1994) discussed the variety of organizational behavior from the perspective of ideology and of ideological beliefs, in other words, the influence of individuals’ personal beliefs in culture and organizational processes.

In addition, individuals have different behavioral styles, values and interests. These characteristics influence how individuals and organizations react to changes in the organizational environment (Kilpatrick, 1996; Shelton et al., 2002), denoting what Kahn and Kram (1994) called internalized aspects and models of authority, that can be of dependence, counter-dependency or interdependence. The psychological aspects, already discussed in the second and third subperiods, are constant in discussions about the world of work, with an increasingly expressive number of studies on this theme, mainly due to its close relationship with organizational behaviors and forms of cultural appropriation. In the 1950s, organizations demanded decision making rationally. In the 1980s, it started to demand information processing. In this period, the emotional demonstration in the organization was understood as weakness and fragility. However, these organizational contexts restricted to the necessary positions, tasks, knowledge and skills, come to be considered “a rich arena for the manifestation of human emotions, both positive and negative” (Muchinsky, 2000). Thus, studies on dysfunctional organizational behavior and culture are also carried out (Balthazard et al., 2006; Goldman et al., 2006). One of the motivations for these changes was the increase in workers’ sickness rates and the costs generated for organizations, which began to develop innovative methods of managing health, workforce and productivity (Amick et al., 2000). Since 2000, the increase in the number of works related to behavior has led the American Psychological Association (APA) to define the years 2000 to 2010 as the “Decade of Behavior” (Geller, 2003).

Related to these issues is job satisfaction, which “is among the most popular and widely debated topics in the areas of organizational behavior and human resource management” (Zeffane, 1994). Judge et al. (2017) also pointed out that job satisfaction is the most researched topic in 100 years of study on I-O Psychology, since terms related to satisfaction appear in more than 70% of studies. However, Wright and Doherty (1998) mentioned that several studies fail to demonstrate a definitive relationship between satisfaction and performance because they consider satisfaction as a synonym for happiness. For the authors, happiness would be a broad state of emotional well-being, while satisfaction refers to an attitude and how the individual cognitively evaluates work-related objects. Therefore, the individual can consider himself happy despite not being satisfied with the work, and conversely. In this perspective, Fisher (2010) mentioned that a “comprehensive measure of individual-level happiness might include work engagement, job satisfaction, and affective organizational commitment,” being necessary to evaluate at various levels, such as transient experiences, stable attitudes and collective attitudes. In 2007, Pfeffer (2007) pointed to the peak of dissatisfaction and feelings of distrust at work, generating negative consequences for both worker and organization. For the author, management and job satisfaction are significant predictors of various dimensions of organizational performance. With regard to the management of human resources, the weaknesses and difficulties of implementation and use of theoretical knowledge culminate in practices that are ineffective and economically expensive (Pfeffer, 2007).

Some studies focus on the discussion of human capital and, more specifically, psychological capital (Luthans and Youssef, 2007; Luthans et al., 2008; Avey et al., 2009, 2011; Dawkins et al., 2013; Abbas et al., 2014; Yu et al., 2019), and referring to the individual motivational propensities that represent positive psychological constructions, such as effectiveness, optimism, hope and resilience (Luthans et al., 2007). In this perspective, De Hoogh and Den Hartog (2008) debate the social responsibility of the leader, the different aspects of ethical leadership and despotic leadership, and the relationships between leadership types and effectiveness and optimism. Thus, the psychological factors associated with emotional intelligence (Rafaeli and Worline, 2001; Ashkanasy and Daus, 2005; Latif et al., 2017), satisfaction and the place of work in the individual’s life are discussed. Blustein (2008) defends the centrality of work for the development and maintenance of psychological life because it can promote the connection between social and economic aspects and provide satisfaction and personal fulfillment. At the same time, work can be synonymous with suffering, which “may be the result of interpersonal and/or collective pressures” (Robert, 2016) with the possibility of the Burnout Syndrome and depersonalization, that is related to the organization’s levels of absenteeism and presenteeism (Demerouti et al., 2009). Judge et al. (2017) analyzed 100 years of research on attitudes and behaviors in organizational environments and concluded that the methods and the use of theoretical knowledge have shown greater sophistication and scientific rigor in recent years, making the field more restricted. On the other hand, the Fourth Industrial Revolution caused an increase in the automation and digitization of work processes (Hirschi, 2018), and political and economic changes that require adjustments in the traditional theoretical paradigm used to understand labor relations (Chernyak-Hai and Rabenu, 2018).

## Conclusion

In this work, we present the evolutionary map and the strategic diagram of 100 years of the field of WOP, through a BNPA supported by the SciMAT software. Our review covered documents from the Scopus and WoS databases published from 1919 to October 11, 2019. We have identified the most important topics related to WOP over time and their implications for individuals and organizations. The emergence of topics such as organizational commitment, healthcare quality, engagement and innovation highlight the expansion of the field of study and the transformation in the world of work. In this perspective, the multiple crossings that Organizational Psychology faced to become the paradigm we know today stood out. Furthermore, we reflect on the impacts of the insertion of the psychology professional in the work context and its contributions to the understanding of human behavior in the organization, considering the social, cultural, emotional and personality biases of the individual, and understanding the organization as a system imbricated by the values, beliefs and ideals of the individuals who give their lives to the organization. Although we have presented a holistic view of the field of study, this work is limited to presenting only the motor themes of WOP. In this sense, future research can deepen the thematic structure of other strategic themes and explore documents available in other databases such as PubMed, APA PsycArticles, BIREME, BVS-Psi, LILACS, PePSIC, Redalyc, among others. Other works can address the WOP in digital transformation scenarios derived from the fourth industrial revolution for integration between individuals, technologies, and organizations. In summary, this study highlights the growth of the WOP research field and its importance in dealing with challenges related to the world of work.

## Data Availability Statement

The raw data supporting the conclusions of this article will be made available by the authors, without undue reservation.

## Author Contributions

MS and LF designed the study protocol and organized the methodology. MS, MB, LM, MC, and NB performed the analysis of results and wrote the manuscript. MS prepared the manuscript and managed the project. All authors contributed to manuscript revision and approved the final draft.

## Conflict of Interest

The authors declare that the research was conducted in the absence of any commercial or financial relationships that could be construed as a potential conflict of interest.
